# Dynamic nature and prognostic value of the neutrophil-to-lymphocyte ratio in critically ill patients with acute kidney injury on continuous renal replacement therapy: A multicenter cohort study

**DOI:** 10.3389/fmed.2023.1162381

**Published:** 2023-03-28

**Authors:** Hyun Lee Ko, Jiyun Jung, Jangwook Lee, Jeong-Hoon Lim, Dha Woon Im, Yong Chul Kim, Jin Hyuk Paek, Woo Yeong Park, Kyeong Min Kim, Soyoung Lee, Sung Woo Lee, Sung Joon Shin, Dong Ki Kim, Seung Seok Han, Chung Hee Baek, Hyosang Kim, Jae Yoon Park, Tae Hyun Ban, Kipyo Kim

**Affiliations:** ^1^Department of Internal Medicine, Uijeongbu Eulji Medical Center, Eulji University, Gyeonggi-Do, Republic of Korea; ^2^Clinical Trial Center, Dongguk University Ilsan Hospital, Goyang, Republic of Korea; ^3^Research Center for Chronic Disease and Environmental Medicine, Dongguk University College of Medicine, Gyeongju, Republic of Korea; ^4^Department of Internal Medicine, Dongguk University Ilsan Hospital, Goyang, Republic of Korea; ^5^Division of Nephrology, Department of Internal Medicine, School of Medicine, Kyungpook National University, Daegu, Republic of Korea; ^6^Department of Internal Medicine, Seoul National University Hospital, Seoul, Republic of Korea; ^7^Department of Internal Medicine, Keimyung University Dongsan Medical Center, Daegu, Republic of Korea; ^8^Department of Internal Medicine, Daejeon Eulji Medical Center, Eulji University, Daejeon, Republic of Korea; ^9^Department of Internal Medicine, Dongguk University College of Medicine, Gyeongju, Republic of Korea; ^10^Department of Internal Medicine, University of Ulsan College of Medicine, Asan Medical Center, Seoul, Republic of Korea; ^11^Department of Internal Medicine, Eunpyeong St. Mary’s Hospital, College of Medicine, The Catholic University of Korea, Seoul, Republic of Korea; ^12^Department of Internal Medicine, Inha University Hospital, Inha University College of Medicine, Incheon, Republic of Korea

**Keywords:** acute kidney injury, continuous renal replacement therapy, neutrophil-to-lymphocyte ratio, inflammation, biomarker

## Abstract

**Introduction:**

Patients with acute kidney injury (AKI) receiving renal replacement therapy constitute the subgroup of AKI with the highest risk of mortality. Despite recent promising findings on the neutrophil-to-lymphocyte ratio (NLR) in AKI, studies have not yet addressed the clinical implication of the NLR in this population. Therefore, we aimed to examine the prognostic value of NLR in critically ill patients requiring continuous renal replacement therapy (CRRT), especially focusing on temporal changes in NLR.

**Methods:**

We enrolled 1,494 patients with AKI who received CRRT in five university hospitals in Korea between 2006 and 2021. NLR fold changes were calculated as the NLR on each day divided by the NLR value on the first day. We performed a multivariable Cox proportional hazard analysis to assess the association between the NLR fold change and 30-day mortality.

**Results:**

The NLR on day 1 did not differ between survivors and non-survivors; however, the NLR fold change on day 5 was significantly different. The highest quartile of NLR fold change during the first 5 days after CRRT initiation showed a significantly increased risk of death (hazard ratio [HR], 1.65; 95% confidence intervals (CI), 1.27–2.15) compared to the lowest quartile. NLR fold change as a continuous variable was an independent predictor of 30-day mortality (HR, 1.14; 95% CI, 1.05–1.23).

**Conclusion:**

In this study, we demonstrated an independent association between changes in NLR and mortality during the initial phase of CRRT in AKI patients receiving CRRT. Our findings provide evidence for the predictive role of changes in the NLR in this high-risk subgroup of AKI.

## Introduction

Acute kidney injury (AKI) affects approximately 13.3 million people worldwide each year and is strongly associated with serious outcomes such as death, cardiovascular disease, and chronic kidney disease (CKD) progression (1–3). AKI-related mortality was estimated to be 23.9% in a pooled meta-analysis (4). Critically ill patients with AKI are at an even higher risk of death. In intensive care settings, the mortality rate of AKI patients requiring renal replacement therapy (RRT) is reported to reach up to 60% (5, 6). In recent decades, an increasing amount of resources, including continuous renal replacement therapy (CRRT), have been utilized to treat patients with AKI (7). Nonetheless, mortality in critically ill patients with AKI remains high, as shown in recent trials (8, 9). Several biomarkers, such as TIMP-2 × IGFBP7, TNF receptor-I, IL-6, and IL-8, have been investigated and have been shown to be associated with mortality in AKI patients (10, 11). However, such biomarkers have not been validated and standardized and are still far from routine clinical use.

Recently, the neutrophil-to-lymphocyte ratio (NLR) has been highlighted as a potential prognostic biomarker for various diseases. A high NLR has been associated with an increased mortality risk in cardiovascular diseases (12, 13), infections (14), and malignant tumors (15, 16). Neutrophils are representative cells of innate immunity, whereas lymphocytes are the core of adaptive immunity. Therefore, NLR, a proportional measure of one type of white blood cells over the other, might serve as a useful marker of systemic inflammation. Also, this measure is readily available through common laboratory tests.

The NLR has also attracted research interest in AKI. Neutrophils and monocytes mediate the acute phase of the first 24–48 h of AKI (17). Lymphocyte infiltration and production of associated cytokines are major factors in AKI 48 h after ischemic injury (18). This may be the underlying pathophysiology of AKI development, which may affect changes in the NLR. However, most studies on NLR have mainly focused on the diagnostic value of NLR for AKI (19), and data on its association with mortality in patients with AKI are inconsistent (20, 21). Moreover, patients included in related studies were mostly in the early stages of AKI, most of whom did not receive RRT (22, 23). Individuals with severe AKI requiring RRT often suffer from multiorgan failure and severe systemic inflammation, which leads to high mortality. Thus, there is an unmet need for potential biomarkers in this population. The clinical implications of NLR in patients receiving CRRT have not been extensively assessed. Furthermore, many previous studies have utilized NLR values at a single time point, and the effect of dynamic changes in NLR has rarely been studied in AKI. Therefore, we aimed to evaluate the prognostic value and dynamic nature of the NLR in patients with severe AKI requiring CRRT using data from a multicenter retrospective cohort.

## Methods

### Study design and population

We retrospectively enrolled patients aged ≥18 years who received CRRT for ≥24 h for AKI at five university hospitals (Dongguk University Ilsan Hospital, Seoul National University Hospital, Kyungpook National University Chilgok Hospital, Eunpyeong St. Mary’s Hospital, and Inha University Hospital) in Korea. We excluded patients who (1) were diagnosed with end-stage kidney disease or terminal-stage cancer and (2) had missing NLR or covariates. Demographic and biochemical data at baseline were collected from electronic health records (EHR) of each hospital, including the date of admission and discharge, CRRT initiation and termination date, sex, age, cause of AKI (sepsis or non-sepsis), body mass index (BMI), Charlson comorbidity index (CCI), C-reactive protein, hemoglobin, heart rate, respiratory rate, potassium, sodium, blood urea nitrogen (BUN), use of mechanical ventilation, acute physiology and chronic health evaluation (APACHE II) score, and sequential organ failure assessment (SOFA) score (24, 25). The main outcome was 30-day mortality based on EHR. This study was approved by the Institutional Review Boards of Dongguk University Ilsan Hospital (DUIH 2022-06-019-002), Seoul National University Hospital (H-2212-142-1,389), Kyungpook National University Chilgok Hospital (KNUCH 2022–09-020), Eunpyeong St. Mary’s Hospital (PC21RIDI0111), and Inha University Hospital (2022-08-046) and informed consent was waived due to the retrospective nature of the study.

### NLR and NLR fold change

The NLR was calculated as the ratio of neutrophil count to lymphocyte count obtained from the differential count over 5 days after CRRT initiation. The NLR fold change was defined as the ratio of NLRs measured on a given day after CRRT initiation and the CRRT initiation date. For example, the NLR fold change on day 2 was the NLR on day 2 after CRRT initiation divided by the NLR on day 1 (NLR day 2/NLR day 1).

### Statistical analyses

We conducted simple and multiple logistic regression to identify the odds ratios of the NLR fold change on 30-day mortality according to different durations after CRRT initiation using c-statistics. Model 1 was a crude model, and Model 2 was additionally adjusted for sex, age, BMI, CCI, sepsis, hypertension, diabetes, and baseline NLR. Model 3 was further adjusted for C-reactive protein, systolic blood pressure, diastolic blood pressure, creatinine, hemoglobin, heart rate, respiratory rate, potassium, sodium, BUN, SOFA score, mechanical ventilation, and CRRT duration. After selecting the fold change on day 5 after CRRT initiation, we estimated the survival probability between 30-day mortality and quartiles of fold change on day 5 in the Kaplan–Meier plot. In addition, a Cox proportional hazards model was used to investigate the association between 30-day mortality and fold change on day 5. The fold change on day 5 was applied as a continuous and categorical variable in Models 1, 2, and 3. In addition, hazard ratios (HR) and 95% confidence intervals (CI) per 1-fold increase are presented in the continuous model, and the HRs of each quartile compared to the lowest quartile were shown in the categorical model. To assess the susceptible subgroups, stratified analyses were conducted for AKI cause (sepsis, non-sepsis), sex, age (<65 years, ≥65 years), and days from admission to CRRT initiation (<3 days and ≥3 days). All statistical analyses were performed using R version 4.2.1 (R Foundation for Statistical Computing, Vienna, Austria).

## Results

### Baseline characteristics

A total of 1,494 patients were included in the main analysis. The mean age was 65.7 years, and 61.8% of the patients were men (Table 1). Hypertension and diabetes mellitus were present in 35.5 and 36.5% of the patients, respectively. The mean duration of CRRT was 9.0 days; 38.8% of the patients had sepsis, and 72.6% were on mechanical ventilation. The mean baseline NLR was 21.9, which is comparable to the NLR value ranged from 17 to 23 in critical stress and inflammation (26). The mean baseline SOFA and APACHE II scores were 11.5 and 25.4, respectively. The baseline characteristics of the quartile groups of participants according to the NLR fold change on day 5 are shown in Table 1. Age, sex, and CCI did not differ among NLR fold-change quartiles. The baseline NLR value was lowest in the fourth quartile of the NLR fold change. The proportion of mechanical ventilation, APACHE II, and SOFA scores were significantly higher in the upper quartiles of NLR fold change on day 5, while no differences were found between the quartiles of baseline NLR value (Supplementary Table S1).

**Table 1:** Baseline characteristics of enrolled participants.

	Total (*n* = 1,494)	Quartiles of NLR fold change on day 5 after CRRT initiation				*p*-value
		Q1 [0–0.52] (*n* = 374)	Q2 [0.52–0.96] (*n* = 373)	Q3 [0.96–1.70] (*n* = 373)	Q4 [1.70–4.63] (*n* = 374)	
Male sex, *n* (%)	924 (61.8)	222 (59.4)	223 (59.8)	239 (64.1)	240 (64.2)	0.348
Age, mean (SD)	65.7 (15.1)	65.4 (15.9)	66.8 (14.9)	65.2 (14.2)	65.5 (15.3)	0.465
Sepsis, *n* (%)	579 (38.8)	161 (43.0)	135 (36.2)	132 (35.4)	151 (40.4)	0.105
BMI, mean (SD)	23.2 (4.4)	23.3 (4.2)	23.4 (4.6)	22.9 (4.2)	23.4 (4.5)	0.335
CCI, mean (SD)	3.5 (2.7)	3.7 (2.7)	3.5 (2.6)	3.5 (2.8)	3.3 (2.7)	0.200
Hypertension, *n* (%)	531 (35.5)	139 (37.2)	148 (39.7)	119 (31.9)	125 (33.4)	0.107
Diabetes, *n* (%)	546 (36.5)	158 (42.2)	132 (35.4)	134 (35.9)	122 (32.6)	0.046
**Biochemical data on CRRT initiation, mean (SD)**						
Neutrophil-to-lymphocyte ratio	21.9 (26.0)	37.9 (41.6)	20.8 (15.6)	17.0 (15.3)	11.9 (10.6)	<0.001
C-reactive protein (mg/L)	12.3 (10.5)	13.2 (10.8)	12.6 (10.1)	11.6 (10.4)	11.9 (10.4)	0.172
Systolic blood pressure (mmHg)	117.1 (26.7)	117.5 (26.4)	119.8 (27.8)	116.3 (27.0)	115.0 (25.5)	0.094
Diastolic blood pressure (mmHg)	60.8 (15.7)	61.2 (14.5)	61.8 (17.2)	60.8 (15.4)	59.5 (15.7)	0.213
Creatinine (mg/dL)	3.1 (2.2)	3.2 (2.2)	3.3 (2.4)	3.0 (2.1)	3.0 (2.2)	0.271
Hemoglobin (g/dL)	9.6 (2.1)	9.6 (2.0)	9.7 (2.2)	9.5 (2.2)	9.5 (2.2)	0.678
Heart rate (bpm)	100.6 (24.6)	100.5 (24.6)	98.8 (24.0)	101.2 (24.3)	102.0 (25.4)	0.332
Respiratory rate (bmp)	22.8 (7.9)	22.7 (7.2)	22.5 (8.6)	22.8 (7.0)	23.2 (8.5)	0.618
Potassium (mEq/L)	4.5 (1.0)	4.4 (1.0)	4.4 (1.0)	4.5 (1.0)	4.5 (1.1)	0.023
Sodium (mEq/L)	136.3 (7.6)	135.8 (7.3)	135.7 (7.3)	136.8 (7.5)	136.8 (8.1)	0.067
Blood urea nitrogen (mg/dL)	56.2 (33.6)	56.3 (36.0)	55.4 (30.5)	58.2 (34.6)	54.8 (32.9)	0.547
**Severity score, mean (SD)**						
SOFA	11.5 (3.4)	11.2 (3.6)	11.3 (3.2)	11.5 (3.4)	12.0 (3.4)	0.011
APACHE II	25.4 (7.6)	24.6 (7.7)	24.9 (7.1)	25.4 (7.7)	26.8 (7.7)	0.001
**CRRT settings, mean (SD)**						
Blood flow rate (mL/min)	111.1 (24.8)	112.7 (24.7)	110.2 (23.6)	109.8 (24.4)	111.6 (26.3)	0.369
Dialysate flow rate (mL/h)	1193.1 (461.0)	1202.7 (480.9)	1183.3 (457.1)	1171.6 (432.6)	1214.9 (472.5)	0.580
Replacement flow rate (mL/h)	942.3 (614.4)	956.8 (647.3)	918.1 (610.9)	929.4 (581.5)	964.7 (617.4)	0.696
CRRT duration (days), mean (SD)	9.0 (14.3)	6.6 (11.0)	8.4 (11.6)	10.6 (17.5)	10.4 (15.6)	<0.001
Mechanical ventilation, *n* (%)	1,084 (72.6)	243 (65.0)	265 (71.0)	281 (75.3)	295 (78.9)	<0.001

BMI, body mass index; CCI, Charlson Comorbidity Index; CRRT, continuous renal replacement therapy; SOFA, Sequential Organ Failure Assessment; APACHE II, Acute Physiology and Chronic Health Evaluation II.

### Dynamic nature and fold change of the NLR

**Figure 1:**
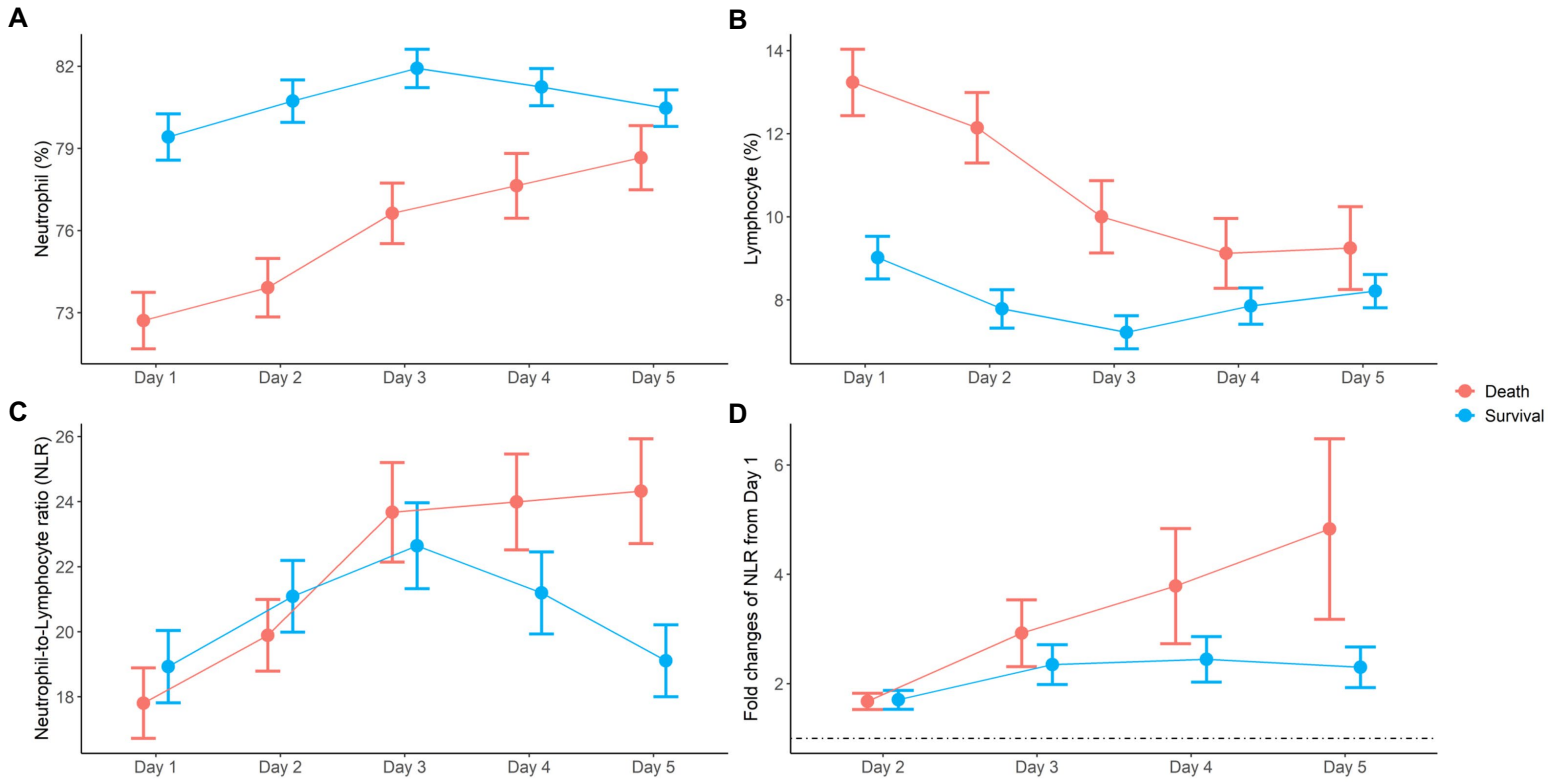
Dynamic changes of neutrophil **(A)**, lymphocyte **(B)**, neutrophil-to-lymphocyte ratio (NLR) **(C)**, and fold changes of NLR **(D)** during the first 5 days after CRRT initiation.

**Figure 2:**
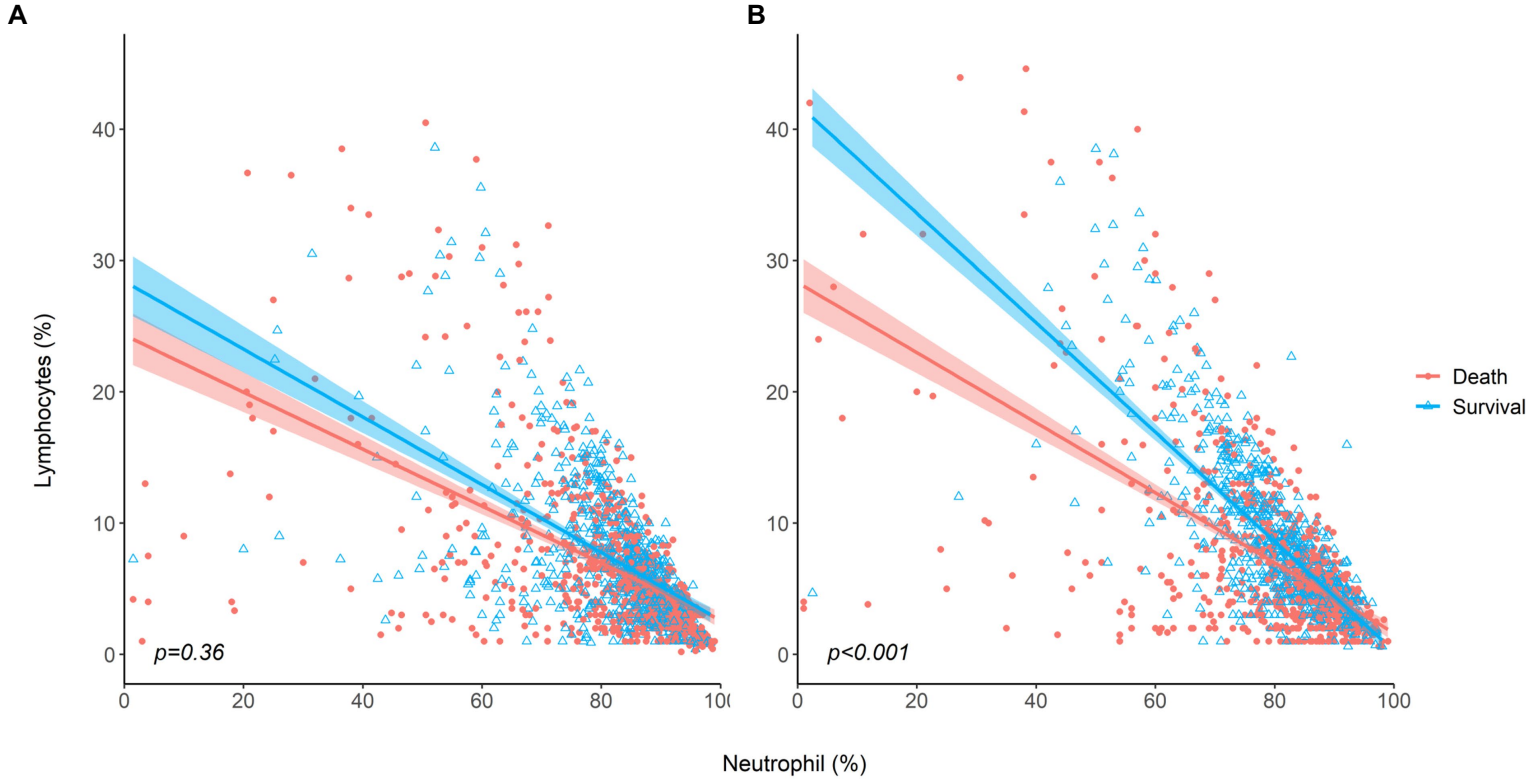
The relationship between neutrophil and lymphocyte counts for survivors and non-survivors **(A)** at baseline, **(B)** on day 5 after CRRT initiation.

Dynamic changes in neutrophils and lymphocytes after CRRT initiation are shown in Figure 1. Overall changes in neutrophil and lymphocyte counts were more obvious in non-survivors than in survivors. The non-survivor group showed increasing neutrophil counts and decreasing lymphocyte counts during the observation period. In terms of the NLR fold change, NLR values in the survivor group started to decrease on day 4 and returned to almost baseline levels on day 5, but this was not observed in the case of non-survivors. The NLR fold change kept increasing in the non-survivor group over the treatment period. The NLR fold change was not different between the survivor and non-survivor groups on day 1, but a significant difference was found on day 5 (*p* = 0.001). When the relationship between neutrophil and lymphocyte counts was plotted and fitted to a linear model according to 30-day mortality, a significant interaction with mortality was found on day 5 but not on day 1 (Figure 2).

### Association between NLR fold change and 30-day mortality

**Figure 3:**
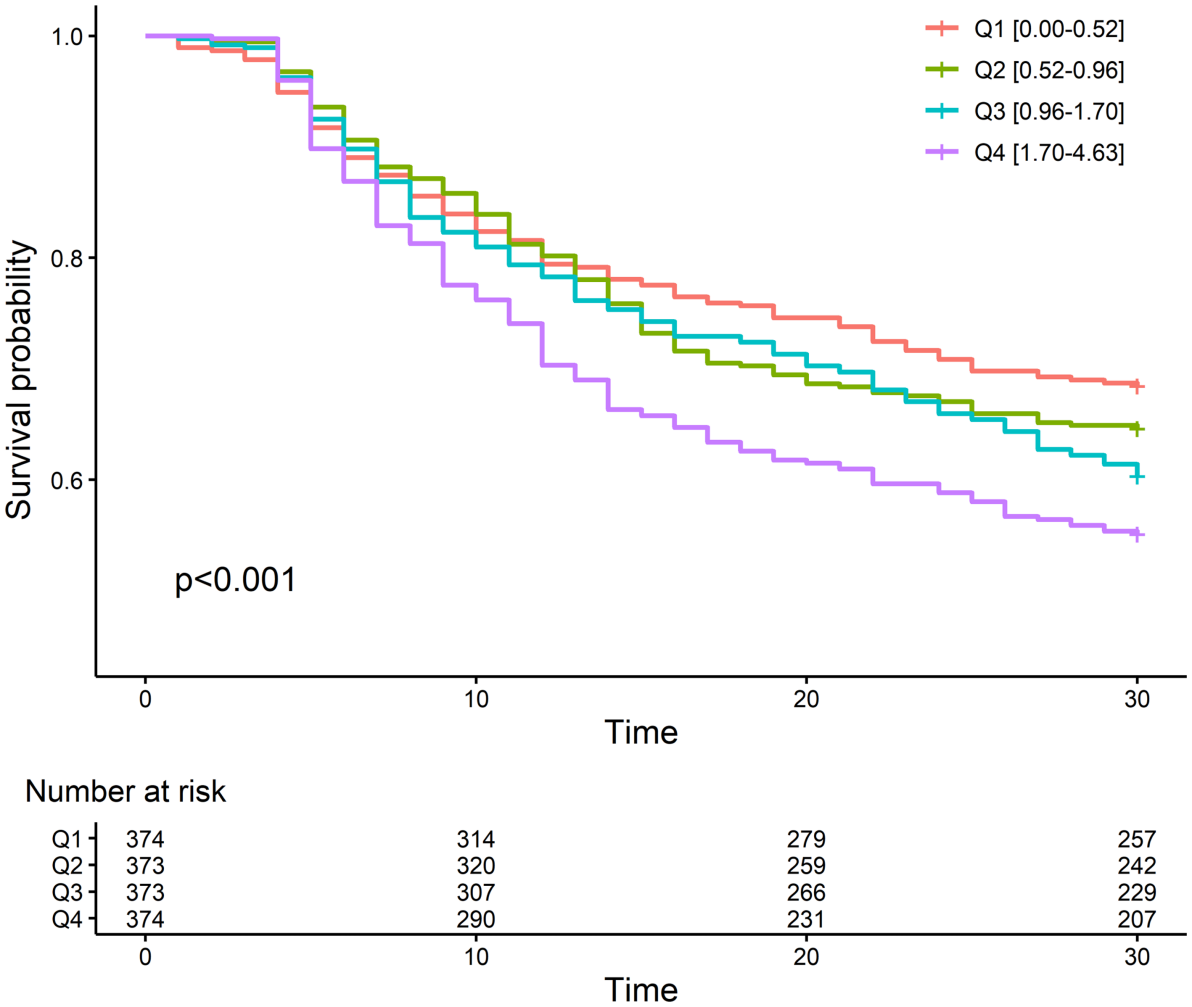
Kaplan–Meier plot between mortality and quartiles of fold change on day 5 after CRRT initiation.

The 30-day mortality rate was 37.9%, which is comparable to that of another study performed on the Korean population (27). The mortality rate was lowest in the first quartile of NLR fold change (31.6%) and highest in the fourth quartile (44.9%). The odd ratios of NLR fold change for different durations after CRRT initiation for 30-day mortality are given in Supplementary Table S2. The NLR fold change was statistically significant in the crude model (Model 1) on day 4 (OR, 1.08; 95% CI, 1.01–1.15) and day 5 (OR, 1.22; 95% CI, 1.10–1.35), but only on day 5 (OR, 1.23; 95% CI, 1.09–1.39) in the fully adjusted model (Model 3). In addition, c-statistic, which indicates the discriminative ability based on sensitivity and specificity, was the highest on day 5. The Kaplan–Meier survival curve of NLR fold change on day 5 for 30-day mortality revealed a significant difference between the NLR fold-change quartiles (Figure 3). The fourth quartile of NLR fold change showed the lowest survival, and the first quartile showed the best survival. In Cox proportional hazard regression analysis, the NLR fold change on day 5 was significantly associated with 30-day mortality (Table 2). The HRs (95% CI) of the second, third, and fourth quartiles were 1.33 (1.02–1.72), 1.42 (1.10–1.85), and 1.65 (1.27–2.15), respectively, with a statistically significant linear trend (*p* for trend <0.001). The HR for each 1-fold increase in NLR fold change was 1.14 (95% CI, 1.05–1.23) in the fully adjusted model.

**Table 2:** Hazard ratio and 95% confidence interval on 30-day mortality associated with the fold change in NLR on day 5 after CRRT initiation.

		Model 1	Model 2	Model 3
Quantile	Q1 [0–0.52]	1 [reference]	1 [reference]	1 [reference]
	Q2 [0.52–0.96]	1.14 (0.89, 1.46)	1.30 (1.00, 1.68)	1.33 (1.02, 1.72)
	Q3 [0.96–1.70]	1.29 (1.01, 1.64)	1.50 (1.16, 1.94)	1.42 (1.10, 1.85)
	Q4 [1.70–4.63]	1.56 (1.23, 1.98)	1.87 (1.45, 2.41)	1.65 (1.27, 2.15)
*p* for trend		<0.001	<0.001	<0.001
Linear		1.16 (1.08, 1.26)	1.21 (1.12, 1.32)	1.14 (1.05, 1.23)

Model 1 was a crude model; Model 2 was additionally adjusted for sex, age, body mass index, Charlson Comorbidity Index, baseline neutrophil-to-lymphocyte ratio, hypertension, diabetes, and sepsis; and Model 3 was further adjusted for C-reactive protein, systolic blood pressure, diastolic blood pressure, creatinine, hemoglobin, heart rate, respiratory rate, potassium, sodium, blood urea nitrogen, SOFA score, mechanical ventilation, and CRRT duration.

### Subgroup analysis

**Figure 4:**
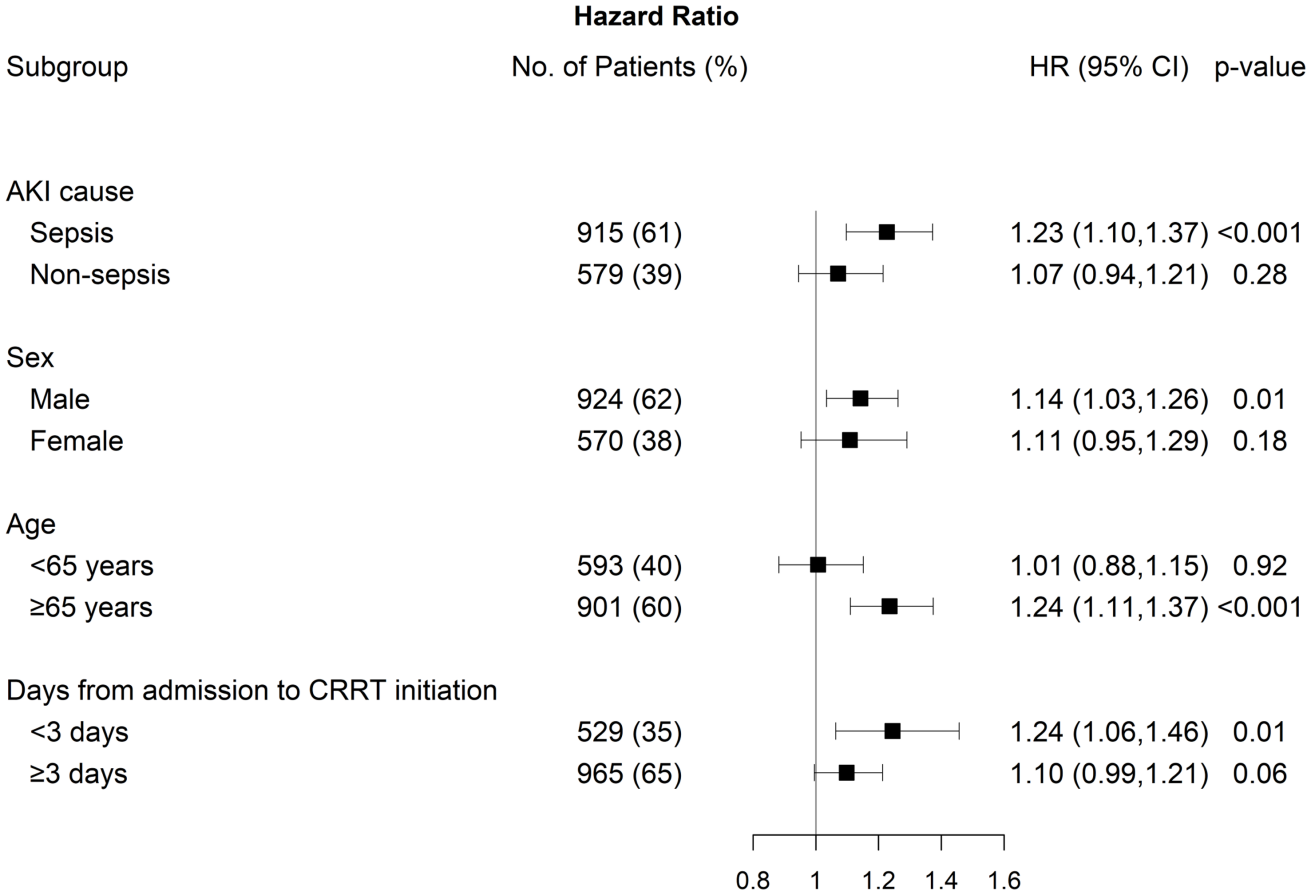
Subgroup analysis between 30-day mortality and fold change on day 5 after CRRT initiation by AKI cause, sex, age, and days from admission to CRRT initiation.

We conducted subgroup analyses by stratifying patients according to AKI cause, sex, age, and the number of days from CRRT initiation (Figure 4). The association between NLR fold change on day 5 and 30-day mortality was statistically significant, particularly in individuals with septic-AKI, male sex, age ≥65 years, and duration between admission and CRRT initiation <3 days. However, no significant modifying effect was identified, except for age (*p* for interaction = 0.04).

## Discussion

Patients with AKI receiving CRRT are a high-risk group and show substantially higher mortality. In this study, we elucidated the dynamic characteristics of NLR during CRRT and showed the associations between changes in NLR and mortality. We identified that changes in NLR during the initial phase after CRRT initiation were markedly different between survivors and non-survivors. In particular, the NLR fold change during the first 5 days after CRRT initiation was significantly associated with 30-day mortality. These results were consistent after adjusting for multiple covariates, including baseline NLR values, showing the importance of continuous monitoring of changes in NLR.

The NLR can be easily calculated using a simple ratio of two immune cell counts. Nonetheless, NLR potentially implicates an immune-inflammatory response in various pathological conditions (26). AKI has a multifactorial pathophysiology, among which inflammation plays a pivotal role in the development of AKI (28, 29). This suggests that the NLR can be used as a useful marker of AKI progression and mortality. In the field of AKI, compared with studies on the diagnostic value of NLR, relatively fewer studies have been conducted on the prognostic value of NLR (19). Previous studies have mainly investigated mortality as a secondary outcome, and none have specifically evaluated patients receiving RRT. A study by Fan et al. using the Medical Information Mart for Intensive Care Database III showed that NLR measured within the first 24 h after admission was independently associated with 30-day and in-hospital mortality in critically ill patients (30). In this study, only 8.9% of the patients received RRT, and 40% had stage 1 and 2 AKI. A retrospective multicenter study in Taiwan also revealed a J-shaped association between NLR near AKI identification and AKI progression, RRT, and in-hospital mortality (22). Despite the positive findings, patients with stage 3 AKI were excluded due to the study design. In AKI patients undergoing RRT, the association between NLR and mortality and its clinical significance have not yet been elucidated. In our study, all participants were critically ill patients with hemodynamic instability who started CRRT at baseline. We revealed the potential predictive value of the NLR in this population. Given the lack of accurate and valid predictive markers, our findings provide valuable insights into NLR in the high-risk population.

Notably, some other studies with similar designs failed to reveal the association of NLR with mortality (20, 21). The causes of this discrepancy could have resulted from the use of baseline NLR only. Still, studies on AKI have focused on the baseline NLR without considering changes in NLR during the clinical course. However, immunologic responses in critically ill patients vary dynamically and immediately, and various immune cells and cytokines are involved in organ failure in real-time. The NLR reflects these highly dynamic immune responses. Indeed, NLR is an earlier marker than the usual inflammatory markers, such as white blood cell count and C-reactive protein levels (31). Recent studies have emphasized the serial assessment of NLR. Riche et al. revealed that, in addition to the baseline NLR, NLR changes for the first 5 days were significantly associated with ICU mortality in patients with septic shock (32). In patients hospitalized with pneumonia, the baseline NLR did not differ between survivors and non-survivors, but incremental changes in the NLR from day 1 to 4 accurately predicted 30-day mortality (33). In our study, NLR at CRRT initiation did not differ between survivors and non-survivors, but a close association was found between mortality and fold changes in NLR rather than the baseline NLR. Similar to the studies evaluating changes in NLR, our observation period for tracking NLR was 5 days.

A high NLR is attributed to neutrophilia and lymphopenia, which are caused by the biological processes of two immune cells in the opposite direction. The currently known mechanisms of neutrophilia and lymphopenia include demargination and margination to the vascular endothelium, delayed and accelerated apoptosis, mobilization and demobilization from the bone marrow, and distribution in blood circulation and redistribution in lymphatic tissue (26). In our subgroup analysis, the NLR was strongly associated with 30-day mortality in sepsis-associated AKI. Sepsis has been the most studied condition in relation to NLR and is also the most common etiology of severe AKI (7). Neutrophils are regarded as key drivers of immune dysregulation in sepsis (34). Increased lymphocyte apoptosis is observed in patients with sepsis, which is closely related to mortality (35). Lymphopenia has also been suggested as a single strong predictor of mortality, distinct from the neutrophil count (36). We found that both neutrophil and lymphocyte counts showed different trends during the first few days after CRRT initiation. The relative changes in lymphocytes were greater than those in neutrophils; thus, the difference in NLR appears to be more driven by a decrease in lymphocytes in non-survivors. In addition, subgroup analysis stratified by age revealed the interaction between age and NLR changes. This finding might have been due to immune dysfunction associated with immunosenescence in the elderly. Indeed, in patients with sepsis, non-survivors aged ≥65 years develop markedly prolonged and profound lymphocytopenia, contributing to delayed death (37).

The potential role of CRRT in the regulation of immune homeostasis remains largely unknown. We cannot assess the effect of CRRT on immune modulation with our retrospective data due to uncontrolled biases and the absence of a control group. Nevertheless, there is indirect evidence for the effects of CRRT on inflammatory immune responses. Murugan et al. reported that intensive RRT significantly lowered plasma IL-6, MIF, and TNFR-I concentrations in patients with initially higher biomarker concentrations (10). A recent study in critically ill patients found that hemodiafiltration resulted in a greater reduction in NLR after the first session than that by hemodialysis, suggesting an immunomodulatory effect of convective therapy (38). However, regardless of the immunomodulatory effects of RRT, monitoring changes in NLR after CRRT initiation may help predict patient outcome, evaluate treatment response, and make follow-up decisions.

Our study has several strengths. First, to the best of our knowledge, we first evaluated the prognostic value of NLR specifically in AKI patients requiring RRT, which is the subgroup with the highest risk of AKI. To date, there are no validated or accurate risk prediction tools for this patient group (39). Second, in contrast to previous studies, we focused on temporal changes in NLR during the initial phase of CRRT and demonstrated their association with adverse outcomes. Third, our study was based on data collected from a relatively large multicenter cohort. However, our study had some limitations. We analyzed retrospective data; thus, prospective trials are required to confirm our findings. This study was based on the Korean population, which may not be generalizable to all patients undergoing CRRT. In the main analysis, patients who died before day 5 were excluded due to missing NLR, which might also have introduced biases. In addition, NLR is not a disease-specific marker and could be affected by various factors such as age, sex, and other comorbidities (31).

In conclusion, we demonstrated that temporal changes in NLR were independently associated with 30-day mortality in AKI patients receiving CRRT. We identified significantly different dynamic natures in neutrophil and lymphocyte counts between survivors and non-survivors. Our results provide evidence for the predictive role of NLR changes during the initial phase of CRRT on mortality and highlight the importance of continuous monitoring of the NLR.

## Data availability statement

The original contributions presented in the study are included in the article/Supplementary material, further inquiries can be directed to the corresponding author/s.

## Ethics statement

The studies involving human participants were reviewed and approved by Institutional Review Boards of Dongguk University Ilsan Hospital (DUIH 2022-06-019-002), Seoul National University Hospital (H-2212-142-1389), Kyungpook National University Chilgok Hospital (KNUCH 2022-09-020), Eunpyeong St. Mary’s Hospital (PC21RIDI0111), and Inha University Hospital (2022-08-046). Written informed consent for participation was not required for this study in accordance with the national legislation and the institutional requirements.

## Author contributions

HLK and KK drafted the manuscript. JJ performed the statistical analysis. JL, J-HL, DI, YK, JHP, WP, KMK, SL, SWL, SS, DK, SH, CB, HK, and JYP contributed to data collection, curation, and interpretation. TB and KK contributed to conception and design of the study and supervised the work. All authors contributed to the article and approved the submitted version.

## Funding

This work was supported by INHA UNIVERSITY Research Grant and Basic Science Research Program through the National Research Foundation of Korea (NRF) funded by the Ministry of Education (2021R1I1A3052012).

## Conflict of interest

The authors declare that the research was conducted in the absence of any commercial or financial relationships that could be construed as a potential conflict of interest.

## Publisher’s note

All claims expressed in this article are solely those of the authors and do not necessarily represent those of their affiliated organizations, or those of the publisher, the editors and the reviewers. Any product that may be evaluated in this article, or claim that may be made by its manufacturer, is not guaranteed or endorsed by the publisher.
