# Supplementary material for: Dynamic nature and prognostic value of the neutrophil-to-lymphocyte ratio in critically ill patients with acute kidney injury on continuous renal replacement therapy: A multicenter cohort study
Source: Front Med (Lausanne). 2023 Mar 28;10:1162381. doi: 10.3389/fmed.2023.1162381 (PMC10086237; doi:10.3389/fmed.2023.1162381)
Supplement: Supplementary file 1 [file Table_1.DOCX]

Supplementary Material

Dynamic nature and prognostic value of the neutrophil-to-lymphocyte ratio in critically ill patients with acute kidney injury on continuous renal replacement therapy: a multicenter cohort study

**Hyun Lee Ko^1,¶^, Jiyun Jung^2,3,¶^, Jangwook Lee^3,4^, Jeong-Hoon Lim^5^, Dha Woon Im^6^, Yong Chul Kim^6^, Jin Hyuk Paek^7^, Woo Yeong Park^7^, Kyeongmin Kim^8^, Soyoung Lee^8^, Sung Woo Lee^1^, Sung Joon Shin^3,4,9^, Dong Ki Kim^6^, Seung Seok Han^6^, Chung Hee Baek^10^, Hyosang Kim^10^, Jae Yoon Park^3,4,9^, Tae Hyun Ban^11,^*, Kipyo Kim^12,^***

*** Correspondence:**Tae Hyun Ban MD, PhD
Division of Nephrology, Department of Internal Medicine, Eunpyeong St. Mary’s Hospital, College of Medicine, The Catholic University of Korea, Seoul, South Korea
1021 Tongil-ro, Eunpyeong-gu, Seoul 03312, South Korea.
Email: [deux0123@catholic.ac.kr](mailto:deux0123@catholic.ac.kr)
Tel: +82- 2-2030-4356, Fax: +82-2-2030-4641

AND

Kipyo Kim, MD, PhD
Division of Nephrology and Hypertension, Department of Internal Medicine, Inha University Hospital, Inha University College of Medicine, Incheon, South Korea
27 Inhangro, Jung-gu, Incheon 22332, South Korea
E-mail: [kpkidney@inha.ac.kr](mailto:kpkidney@inha.ac.kr)
Tel: +82-32-890-3246

**Supplementary Table 1. Baseline characteristics according to the quartiles of NLR on day 1.**

|  | Quartiles of NLR on day 1 | | | |  |  |
| --- | --- | --- | --- | --- | --- | --- |
|  | Q1 [0–8.4] (n=374) | Q2 [8.4–14.8] (n=373) | Q3 [14.8–26.7] (n=373) | Q4 [26.4–467] (n=374) | *P*-value |  |
|  |  |  |  |  |  |  |
| Male sex, n (%) | 241 (64.4) | 214 (57.4) | 238 (63.8) | 231 (61.8) | 0.184 |  |
| Age, mean (SD) | 65.0 (15.2) | 65.7 (15.3) | 66.7 (14.3) | 65.6 (15.4) | 0.494 |  |
| Sepsis, n (%) | 144 (38.5) | 131 (35.1) | 147 (39.4) | 157 (42.0) | 0.285 |  |
| BMI, mean (SD) | 23.4 (4.2) | 24.0 (4.6) | 22.8 (4.3) | 22.7 (4.2) | <0.001 |  |
| CCI, mean (SD) | 3.4 (2.7) | 3.3 (2.6) | 3.6 (2.7) | 3.7 (2.9) | 0.315 |  |
| Hypertension, n (%) | 131 (35.0) | 149 (39.9) | 128 (34.3) | 123 (32.9) | 0.204 |  |
| Diabetes, n (%) | 136 (36.4) | 126 (33.8) | 145 (38.9) | 139 (37.2) | 0.538 |  |
| Biochemical data, mean (SD) |  |  |  |  |  |  |
| C-Reactive Protein (mg/L) | 11.5 (10.9) | 11.8 (10.2) | 12.7 (10.4) | 13.3 (10.2) | 0.096 |  |
| Systolic blood pressure (mmHg) | 117.4 (28.7) | 117.4 (26.4) | 117.6 (26.7) | 116.1 (25.0) | 0.845 |  |
| Diastolic blood pressure (mmHg) | 62.2 (16.2) | 60.7 (15.5) | 60.3 (15.1) | 60.1 (16.1) | 0.266 |  |
| Creatinine (mg/dL) | 3.2 (2.3) | 3.3 (2.7) | 3.0 (1.8) | 3.1 (2.1) | 0.349 |  |
| Hemoglobin (g/dL) | 9.6 (2.2) | 9.8 (2.2) | 9.6 (2.1) | 9.4 (1.9) | 0.057 |  |
| Heart rate (bpm) | 102.3 (25.4) | 100.0 (24.8) | 98.1 (24.4) | 102.1 (23.6) | 0.06 |  |
| Respiratory Rate (bmp) | 23.0 (8.1) | 22.9 (8.7) | 22.5 (7.0) | 22.7 (7.5) | 0.82 |  |
| Potassium (mEq/L) | 4.5 (1.1) | 4.5 (1.1) | 4.4 (0.9) | 4.4 (0.9) | 0.157 |  |
| Sodium (mEq/L) | 137.0 (7.8) | 136.8 (7.6) | 136.9 (7.3) | 134.4 (7.2) | <0.001 |  |
| Blood Urea Nitrogen (mg/dL) | 56.1 (33.7) | 51.5 (31.2) | 55.4 (30.3) | 61.7 (37.8) | 0.001 |  |
| Severity score, mean (SD) |  |  |  |  |  |  |
| SOFA | 11.6 (3.6) | 11.3 (3.4) | 11.5 (3.5) | 11.5 (3.2) | 0.789 |  |
| APACHE Ⅱ | 25.7 (8.0) | 25.4 (7.6) | 25.4 (7.3) | 25.2 (7.6) | 0.829 |  |
| CRRT settings, mean (SD) |  |  |  |  |  |  |
| Blood flow rate (mL/min) | 111.6 (26.3) | 110.2 (24.0) | 110.0 (23.6) | 112.5 (25.2) | 0.484 |  |
| Dialysate flow rate (mL/h) | 1226.3 (508.2) | 1171.2 (434.5) | 1164.5 (457.1) | 1210.2 (438.9) | 0.193 |  |
| Replacement flow rate (mL/h) | 940.3 (661.7) | 940.3 (561.4) | 960.7 (617.2) | 927.8 (614.5) | 0.908 |  |
| CRRT duration (days), mean (SD) | 8.0 (12.4) | 8.4 (11.5) | 10.1 (19.5) | 9.3 (12.1) | 0.19 |  |
| Mechanical ventilation, n (%) | 264 (70.6) | 273 (73.2) | 280 (75.1) | 267 (71.4) | 0.524 |  |

**Supplementary Table 2**. Odds ratio and 95% confidence interval of 30-day mortality associated with increased fold change during the first 5 days after CRRT initiation.

|  |  | Fold change after CRRT initiation | | | |
| --- | --- | --- | --- | --- | --- |
|  |  | Day 2 | Day 3 | Day 4 | Day 5 |
| Model 1 | OR (95% CI) | 1.03 (0.95,1.12) | 1.04 (0.98,1.11) | 1.08 (1.01,1.15) | 1.22 (1.10,1.35) |
|  | c-statistics | 0.49 | 0.51 | 0.54 | 0.56 |
| Model 2 | OR (95% CI) | 1.07 (0.98,1.18) | 1.07 (1.00,1.13) | 1.11 (1.03,1.19) | 1.32 (1.18,1.48) |
|  | c-statistics | 0.60 | 0.60 | 0.61 | 0.63 |
| Model 3 | OR (95% CI) | 1.01 (0.91,1.11) | 1.02 (0.95,1.09) | 1.06 (0.98,1.14) | 1.23 (1.09,1.39) |
|  | c-statistics | 0.72 | 0.72 | 0.72 | 0.73 |

Model 1 was a crude model; Model 2 was additionally adjusted for sex, age, body mass index, Charlson Comorbidity Index, baseline neutrophil-to-lymphocyte ratio, hypertension, diabetes, and sepsis; and Model 3 was further adjusted for C-reactive protein, systolic blood pressure, diastolic blood pressure, creatinine, hemoglobin, heart rate, respiratory rate, potassium, sodium, blood urea nitrogen, SOFA, mechanical ventilation, and CRRT duration.
